# Measurement of the coherent beam properties at the CoSAXS beamline

**DOI:** 10.1107/S1600577521009140

**Published:** 2021-10-05

**Authors:** Maik Kahnt, Konstantin Klementiev, Vahid Haghighat, Clemens Weninger, Tomás S. Plivelic, Ann E. Terry, Alexander Björling

**Affiliations:** a MAX IV Laboratory, Lund University, Fotongatan 2, 224 84 Lund, Sweden

**Keywords:** ptychography, coherence, SAXS, XPCS

## Abstract

The CoSAXS beamline at the MAX IV Laboratory is a modern multi-purpose (coherent) small-angle X-ray scattering (CoSAXS) instrument, designed to provide intense and optionally coherent illumination at the sample position, enabling coherent imaging and speckle contrast techniques. X-ray tracing simulations used to design the beamline optics have predicted a total photon flux of 10^12^–10^13^ photons s^−1^ and a degree of coherence of up to 10% at 7.1 keV. The normalized degree of coherence and the coherent flux of this instrument were experimentally determined using the separability of a ptychographic reconstruction into multiple mutually incoherent modes and thus the *Co*herence in the name *Co*SAXS was verified. How the beamline can be used both for coherent imaging and XPCS measurements, which both heavily rely on the degree of coherence of the beam, was demonstrated. These results are the first experimental quantification of coherence properties in a SAXS instrument at a fourth-generation synchrotron light source.

## Abstract 2

The coherence properties of the beam provided at the CoSAXS beamline at the MAX IV Laboratory are determined experimentally using multi-mode ptychography. The coherent fraction of the beam is then used for a proof-of-concept XPCS measurement, illustrating the beamline’s capabilities.

## 1. Introduction

Fourth-generation synchrotron light sources have been developed to reduce the emittance of the storage rings and thus increase the brilliance of the provided X-ray beams. Many modern techniques rely on this high brilliance as it goes hand in hand with a larger degree of coherence of the beam. The CoSAXS beamline at the MAX IV Laboratory, the first of this new generation of synchrotron light sources, recently came online. It is a multi-purpose small-angle X-ray scattering (SAXS) instrument, designed to provide a high-intensity X-ray probe with a selectable degree of transverse coherence (Plivelic *et al.*, 2019 ▸).

**Figure 1:**
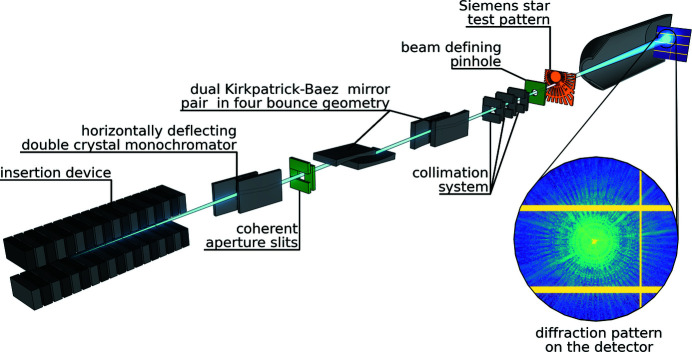
Scheme of the main X-ray optical elements in the experiment. From left to right: in-vacuum undulator, horizontally deflecting double-crystal monochromator (Si 111), coherence aperture slits *S*_CAp_ (in green), two pairs of bendable Kirkpatrick–Baez (KB) mirrors, three slits minimizing parasitic contributions, a pinhole (in green) to limit the beam size, a Siemens star as sample (in orange) and a typical diffraction pattern on the hybrid pixel X-ray detector EIGER2 X 4M detector inside a vacuum vessel.

Figure 1 ▸ shows the main X-ray optical elements built into the beamline. By design there is one optical element, the set of slits which define the coherence aperture *S*_CAp_, which allows control of the transverse degree of coherence. The monochromator upstream of this aperture defines the longitudinal coherence. Two pairs of vertically and horizontally focusing bendable Kirkpatrick–Baez (KB) mirrors (VFMs and HFMs, respectively) control the parallelism or divergence of the beam and thus define the focal position of the beam. All other optical elements are apertures to collimate the beam or reduce the parasitic scattering. The performance of this X-ray optical setup has been simulated using a hybrid ray-tracing and field-propagation approach (Klementiev & Chernikov, 2014 ▸). Using these tools a total photon flux of 10^12^–10^13^ photons s^−1^ in the 4–20 keV energy range and a degree of coherence of up to 10% at 7.1 keV has been predicted in a previous publication (Plivelic *et al.*, 2019 ▸).

Here, we experimentally determine these beam properties at 6.7 keV and compare them against the corresponding simulations. The results show that, while further optimization will be needed to achieve the predicted maximum intensity, the beam fully displays the expected transverse coherence fraction as predicted by the simulations. We show by exemplary measurements that the high-quality beam at the CoSAXS beamline can be successfully used for coherent imaging and X-ray photon correlation spectroscopy (XPCS) measurements of diffusion rates of colloidal particles.

## 2. Results

In the following section three different experiments are reported: (*a*) we determined the profile and phase curvature of the full beam at the sample position using tele-ptychography, (*b*) by repeated ptychographic imaging of a test structure, we quantified the coherence properties of the beam as a function of *S*_CAp_, and finally (*c*) we performed proof-of-concept XPCS measurements on colloidal particles.

### 2.1. Beam size at the sample position

The beam size after coarse adjustment of the focusing mirrors was determined at the sample position using a tele-ptychography measurement (Maiden & Rodenburg, 2009 ▸; Tsai *et al.*, 2016 ▸; Verezhak *et al.*, 2018 ▸, 2021 ▸). In contrast to standard forward ptychography, where a photon beam with limited size illuminates an extended sample, in a tele-ptychography experiment an analyzer, a pinhole of a limited size, is scanned through an extended beam. This technique yields the beam’s wavefront’s relative phases and amplitudes at the position of the analyzer pinhole, as well as the transmission function of the analyzer pinhole, and avoids convolving the measured beam profile with the pinhole shape. In tele-ptychography experiments with a sample, the reconstructed wavefront is usually numerically propagated up­stream to the position of the sample. In this case we did not use a sample, but instead placed the analyzer pinhole right where normally a sample would be placed in the beam. The size of the pinhole (5 µm) fulfilled the sampling condition for the width *w*_*x*,*y*_ of the exit wavefront,

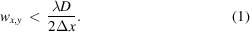

Here, the sample-to-detector distance was *D* = 14.67 m, the detector pixel size was 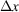 = 75 µm, and the X-ray wavelength λ corresponded to a photon energy of 6.7 keV.

**Figure 2:**
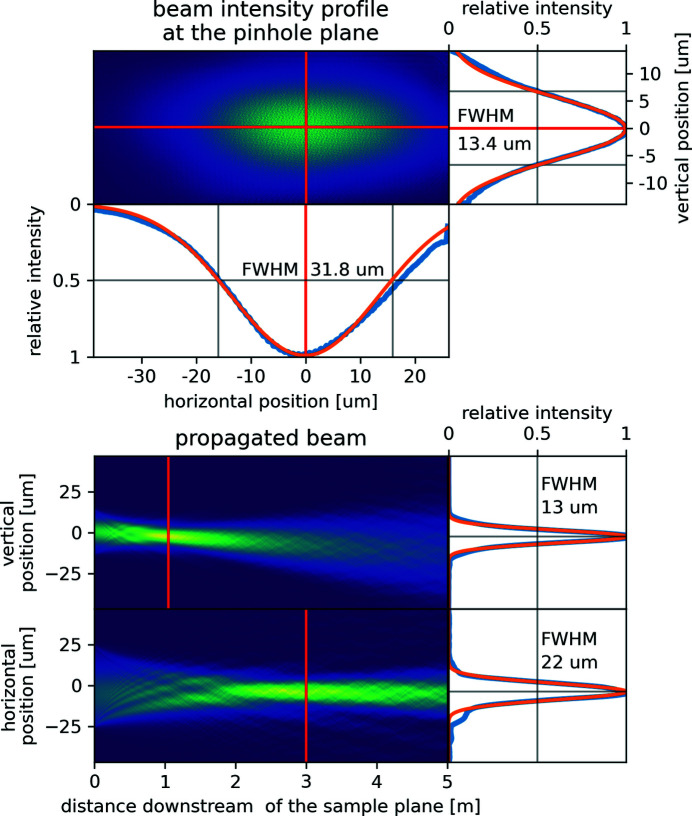
Top: ptychographically reconstructed beam profile at the sample position (here the pinhole took the place of the sample). The extracted vertical and horizontal beam profiles are shown in blue and the fitted Gaussians, from which the full widths at half-maximum (FWHM) of 13.4 µm and 31.8 µm were measured, are shown in orange. Bottom: numerically downstream propagated and projected beam intensity profiles, revealing the relative focus positions and focus sizes (FWHM): 13 µm at 1 m (vertical) and 22 µm at 3 m (horizontal).

The pinhole was scanned in 57 × 20 steps of 1 µm size through the beam, resulting in 58 × 21 = 1218 diffraction patterns. The ptychographic reconstruction was performed using *ptypy* (Enders & Thibault, 2016 ▸) running 1000 iterations of the Difference Map algorithm (Thibault *et al.*, 2009 ▸) followed by 1000 iterations of the Maximum Likelihood algorithm (Thibault & Guizar-Sicairos, 2012 ▸). Vertical and horizontal cuts of the reconstructed beam intensity profile were fitted with Gaussians to extract full width at half-maximum (FWHM) beam sizes of 13.4 µm and 31.8 µm, respectively (see Fig. 2 ▸, upper panel). Numerically propagating the reconstructed beam revealed that both the VFMs and the HFMs were bent to focus the beam downstream of the sample (see Fig. 2 ▸, lower panel). These focal positions are well within the design specifications of the beamline and can be shifted by bending the VFMs and the HFMs to any position between the sample position and 17 m further downstream at the very end of the vacuum vessel. The vertical and horizontal focal spot sizes described in Fig. 2 ▸ (lower panel) are in agreement with X-ray tracing simulations (Plivelic *et al.*, 2019 ▸).

### 2.2. Coherence properties of the beam

The size of the full beam (without any collimation) at the sample position was too large to fulfill equation (1). Adjusting the VFMs and the HFMs to focus at the sample position would not have sufficiently reduced it. Thus a pinhole of diameter 10 µm was placed 17.5 mm upstream of the sample position to crop the beam for all following standard forward ptychographic experiments investigating its coherence properties. From the full beam profile reconstructed in the previous experiment, we estimated that such a pinhole will allow for a 15% transmittance of the full beam intensity.

A Siemens star test pattern (radial spoke holes in 500 nm-thick tantalum) was chosen as sample. Its sharp edges in all directions allow for an uncomplicated reconstruction which can provide detailed information on the illumination field. The sample (placed 14.67 m upstream of the detector) was raster scanned through the beam to record ptychographic data sets. Each scan consisted of 40 × 40 steps of 1 µm step size resulting in 41 × 41 = 1681 recorded diffraction patterns. These measurements were repeated for different values of *S*_CAp_, altering the coherence properties of the beam. The aperture was kept square for all scans. Absorbers (Al foils up to a total thickness of 240 µm) were used and adapted for each slit setting to not saturate the detector with the incoming photon flux as no central beam stop was used.

**Figure 3:**
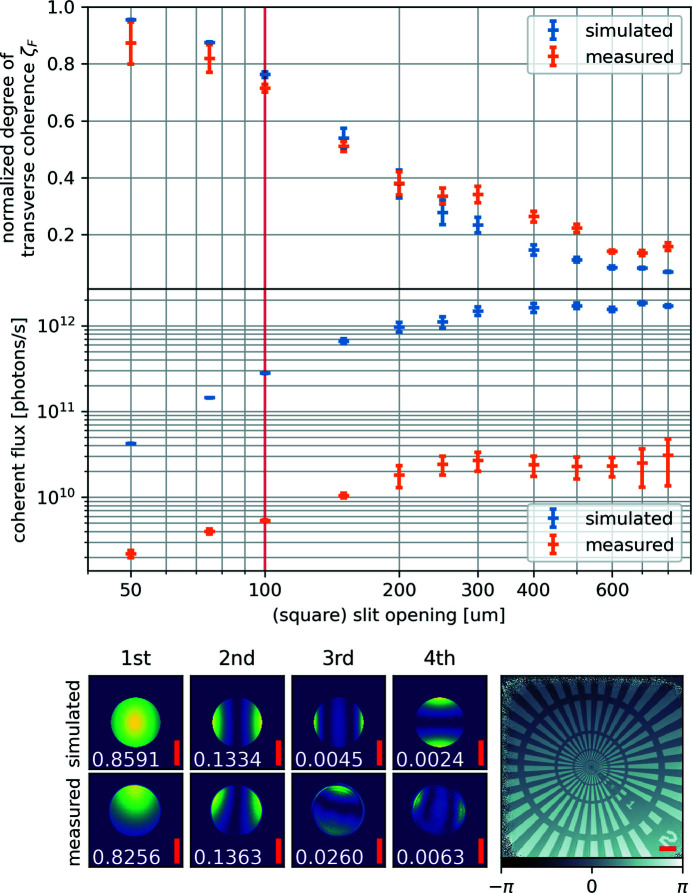
Experimentally determined coherent beam parameters depending on the coherence aperture slit *S*_CAp_ opening. Top: normalized degree of transverse coherence ζ_*F*_ according to equation (2). The error bars show the standard deviation of the fitted transverse coherence ζ_*F*_ estimate. Center: the estimate for coherent flux downstream of the pinhole, in the simulation (blue) and the measurement (orange), assuming that the coherent flux is ζ_*F*_ times the total flux. The vertical red line corresponds to the data shown in the bottom part of the figure: the four strongest modes at the pinhole position and their strengths 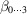 (in white) retrieved from the simulation and the measurement at *S*_CAp_ = 100 µm × 100 µm together with the reconstructed phase of the Siemens star. All scale bars (red) have a length of 5 µm.

To extract the information about the beam’s coherence properties from these data, each scan was reconstructed as described above, but now using 20 mutually incoherent modes (Thibault & Menzel, 2013 ▸) for the probing beam. The diffraction patterns were cropped to a size of 256 × 256 pixels, which resulted in a pixel size of 141 nm in reconstructions. The Siemens star test pattern was faithfully reconstructed for all scans (see example in Fig. 3 ▸). The periodic structures were recovered down to the ring with 200 nm lines and spaces. The reconstructed modes for the probing beam were orthogonalized and sorted by their intensity. The normalized degree of coherence ζ_*F*_ was then retrieved by fitting the decay in strength β_*n*_ of the reconstructed modes relative to the strongest mode β_0_ (Vartanyants & Singer, 2010 ▸; Moxham *et al.*, 2020 ▸), 

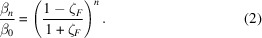

The normalized degree of coherence as a function of the coherence aperture *S*_CAp_ is shown in Fig. 3 ▸ (upper panel). Using the total intensity measured on the detector, the known absorption of the sample, the transmission of 300 mm air path and the known absorber settings, we retrieved the total flux and together with the fitted ζ_*F*_ the coherent flux (downstream of the pinhole) at the time of the experiment. The resulting coherent flux values are shown in Fig. 3 ▸ (center panel).

The experiment was also modeled using X-ray tracing and wave propagation with the *xrt* software package (Klementiev & Chernikov, 2014 ▸; Plivelic *et al.*, 2019 ▸). As in the performed experiment, the photon energy was 6.7 keV and a 10 µm beam-defining pinhole was placed 17.5 mm upstream of the sample position. In contrast to the real experiment, the focus position of the beam was, however, modeled at the sample position. Using the same focus position as in the experiment would have increased the computing time unreasonably (as too many sampling photons would have missed the beam-defining pinhole). Choosing instead the optimal focus position reduced the computing time to a week and only has minimal impact on the relative strengths and shapes of the retrieved beam modes behind the beam defining pinhole.

The strongest 20 of the resulting modes were used to fit ζ_*F*_ according to equation (2). The fitted ζ_*F*_ were then multiplied with the total simulated flux to estimate the coherent flux. The results of these simulations are shown in blue in Fig. 3 ▸.

The simulated and the measured normalized degrees of transverse coherence ζ_*F*_ and the shape of the extracted modes agree very well. The shown errors bars originate from the fit quality of equation (2). The exponential decay of the mode strengths β_*i*_ is based on the model of the beam as separable Gaussians (Vartanyants & Singer, 2010 ▸). Due to the position of the coherence aperture *S*_CAp_ in a divergent beam, as the beamline does not have an intermediate focus (a secondary source), this model fits for very small and very large slit openings, *i.e.* when the beam is either nearly a point source or not cut at all. For intermediate slit openings, the factor between the strength of adjacent modes β_*i*_ and β_*i*+1_ might not be constant, as the Gaussian model is not exactly met. This results in increased error bars of the simulated data points in Fig. 3 ▸ (top panel) for those intermediate slit openings. The measured ζ_*F*_ do not approach as high numbers as the simulated results for the smallest slit openings. This is most likely a result of underlying vibrational modes. As the ptychographic algorithm cannot differentiate between incoherence in the beam and vibration-induced shifts between sample and beam, the presence of vibrations reduces the overall effective degree of coherence. It also changes the relative mode strength, from the expected model of exponential decay, which results in the larger error bars at smaller slit openings.

The bump towards higher measured degrees of coherence around slit openings of 300 µm is an unexpected but real result. The ptychographic scans were performed with successively increasing slit openings. A non-smooth beam profile at the *S*_CAp_ position could explain the observed bump. When opening the slits beyond 250 µm, the increased aperture might not have caught additional intensity in the extra aperture area, which will keep the estimated coherence similar to the previous data point or at least higher than predicted in the simulation. The larger error bars in that region again indicate the limitations of the Gaussian model.

For both the simulation and the measurement, the coherent flux (estimated as ζ_*F*_ times the total flux) increases with larger coherence apertures *S*_CAp_ and levels off at an opening of around 300 µm. The simulation and the measurement differ from each other in intensity by roughly a factor of 20 for the smallest measured openings of *S*_CAp_ and roughly a factor of 45 for the largest measured openings of *S*_CAp_. The reduced values observed experimentally could have multiple sources. One is misalignment between pinhole and beam, caused by slow drift of the table holding sample and pinhole. The off-center positioning can be seen in the strongest measured mode (see Fig. 3 ▸, bottom left), while the drift can be seen when comparing these strongest modes in the order in which the scans were taken (not shown here). The observed pinhole misalignment, found from images of the strongest modes, was at least 5 µm. With the known beam profile, we could estimate that this limits the flux reduction to at least a factor of 1.5. The beam itself was not focused in the plane of the pinhole (as shown in Fig. 2 ▸), which would account for a loss of a third of the total transmitted beam intensity in case of an optimal pinhole placement. The pointing of the electron beam might have been slightly off. The positions of the VFMs and HFMS might have drifted. Also, imperfect pointing of the electron beam as well as small mis-alignments of the VFMs and HFMs might have reduced the flux. The simulations were carried out assuming perfect mirror surfaces, while metrology measurements have revealed both tiny slope errors as well as surface roughness that would both result in a slightly larger focal spot. The shown flux numbers only represent the achieved values during this particular experiment. It can be expected that, in the future, after more beamline commissioning, optimized settings will increase the available flux as it is common for instruments to not perform at peak performance immediately after starting operation.

Even with the flux being lower than expected, the beam needed to be attenuated by a factor of up to 300 for these imaging experiments. The reason for that is the low divergence of the beam delivered at the sample position. Even when propagating 15 m to the detector, the direct beam all ends up in a single pixel. Adding more divergence to the beam by using additional focusing optics would allow using the full flux as well as creating a small enough beam profile to inherently fulfill the over-sampling criterion for ptychographic measurements. This would enable a wide range of imaging methods (ptychography, holography, dark field X-ray microscopy) at the CoSAXS beamline.

This study was performed at one of the most representative photon energies of the CoSAXS beamline, 6.7 keV, where experimental conditions for coherent illumination are most relaxed. It would be desirable to repeat similar measurements at other photon energies throughout the whole energy range of this instrument (4 to 20 keV), but is beyond the scope of the present manuscript. The estimated transverse normalized degree of coherence ζ_*F*_ could also be measured more directly by an experiment based on a Young’s double slit (Lyubomirskiy *et al.*, 2016 ▸) or on a non-redundant array of slits (Westfahl *et al.*, 2017 ▸). The advantage of the ptychographic method presented here is that the retrieved set of coherent modes can be treated the same way as the simulated modes, which allows for a direct comparison.

### 2.3. Proof-of-concept XPCS measurement

Having identified the aperture *S*_CAp_ settings for a beam which is coherent across at least 10 µm in the previous experiment, we proceeded to use the coherent beam for a proof-of-concept X-ray photon correlation spectroscopy (XPCS) experiment (Robert, 2007 ▸). All absorbers, the Siemens star test structure and the 10 µm pinhole were removed from the beam path. The focusing of the beam was not changed. A beam stop was placed in front of the detector to block the direct beam. *S*_CAp_ was closed to 100 µm × 100 µm. A capillary with a diameter of 300 µm was filled with a suspension of nanometric silica-coated hematite ellipsoids (length 300 nm, width 100 nm) (Pal *et al.*, 2018 ▸) in glycerol. The capillary was placed inside the beam path and speckles could be immediately observed on the detector. A time series of the speckle pattern was recorded at the maximum frame rate of the Eiger2 X 4M detector: 500 Hz.

**Figure 4:**
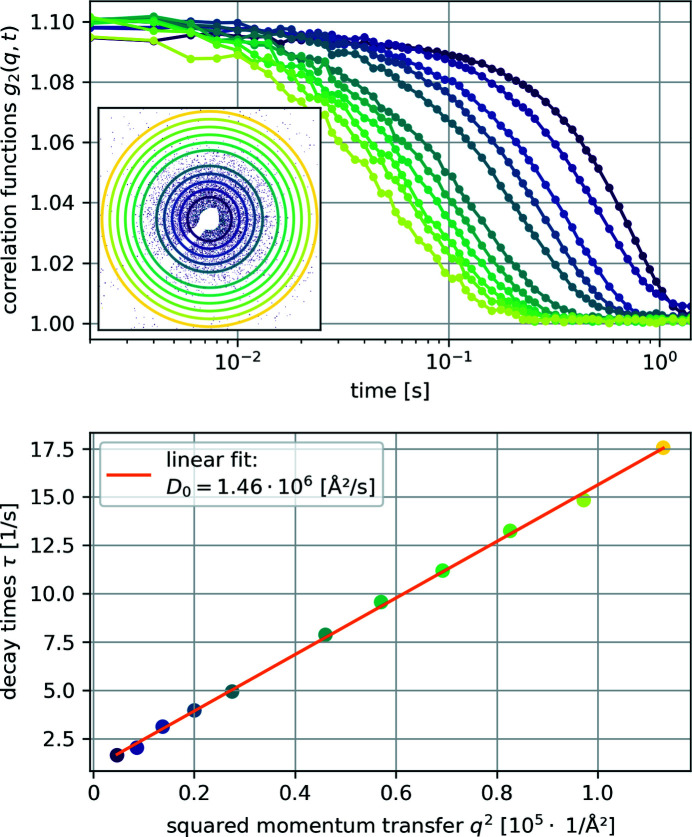
Top: calculated intensity correlation functions *g*_2_(*q*, *t*) from the measurements on silica-coated hematite ellipsoids dispersed in glycerol. The inset shows one of the recorded scattering patterns and the rings mark the *q* used for the other plots. Bottom: fitted decay times τ plotted against *q* ^2^ showing a linear decency and the linear fit to estimate the free particle diffusion coefficient *D*_0_. The color coding of each data point marks the corresponding intensity correlation function *g*_2_(*q*, *t*) and the ring/radius in the inset.

From those recorded speckle patterns the normalized (second-order) intensity correlation function *g*_2_(*q*, *t*) was calculated for multiple momentum transfer regions *q* = 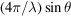 , where 2θ is the scattering angle (see Fig. 4 ▸, top panel). Each of these curves was fitted with a stretched exponential to obtain decay times τ. Plotting these τ against *q* ^2^, the free particle (Stokes–Einstein) diffusion coefficient *D*_0_ was retrieved as the slope of a linear fit to be 1.46 × 10^6^ Å s^−1^ (see Fig. 4 ▸ bottom) (Robert, 2007 ▸).

Using a Stokes–Einstein equation modified for ellipsoids (Edward, 1970 ▸), and the known aspect ratio (*a*/*b* = 3) and particle size, the viscosity η could be calculated as 

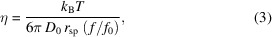

where *k*_B_ is the Boltzmann constant, *T* is the temperature, *r*_sp_ is the radius of a sphere with the same volume as the ellipsoids and *f*/*f*_0_ is the ratio of the frictional coefficients of ellipsoids and spheres of the same volume [for *a*/*b* = 3, this fraction *f*/*f*_0_ is about 1.1 (Perrin, 1936 ▸)]. The resulting viscosity of 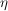 = 0.1852 Pa s is lower than that of pure glycerol at room temperature (1.412 Pa s). The lower estimated value could be a result of either residual water or of the X-ray beam heating the suspension, both of which would lower the viscosity. The estimated viscosity value corresponds to a glycerol–water mixture with a volume fraction of 86% glycerol at room temperature, or to pure glycerol at 46°C (González *et al.*, 2011 ▸), both of which are realistic scenarios. Instabilities of the sample mounting and the beam at the sample position can also not be completely ruled out. Both effects would have lowered the retrieved value for the viscosity.

Using the normalized degree of coherence of the beam, we were, for the first time, able to perform an XPCS measurement at the CoSAXS beamline. Using the Eiger2 X 4M detector at its maximum frame rate of 500 Hz, we measured quantitative decay times τ down to 17.5 s^−1^ at *q* up to 3.36 × 10^−3^ Å^−1^. Improving the experimental conditions by using stronger scattering samples or longer acquisition series, a faster detector and either higher photon energies or shorter detector distances, these values could be pushed to observe faster processes at even smaller length scales. A challenge for similar experiments in the future will be to prevent or quantify the influence of the intense probing beam on the sample.

## 3. Conclusion

We have shown that the use of a transversally scannable pinhole allows measuring the complex beam profile and thus beam divergence and mirror performance at the CoSAXS beamline. To our knowledge it is the first time tele-ptychography has been used to characterize the flat beam itself. This simple tool can be used to easily check the settings of the beamline just prior to an experiment. Using a cropped beam and multi-mode ptychographic reconstructions, we have shown that the coherence aperture *S*_CAp_ can indeed be used to freely vary the beam properties between nearly fully coherent but less intense or high intensity but lower normalized degree of coherence. The experimentally estimated slit settings required for a nearly fully coherent beam agree with simulations. The experimental results for the available flux qualitatively agree with simulations but differ in magnitude. This discrepancy will need further investigation but is likely to be a matter of beamline and experimental optimization. But even the measured available coherent photon flux of the beamline provides conditions not readily available at third-generation sources. Finally we have proven that the CoSAXS beamline is able to perform quantitative XPCS experiments which rely on the degree of coherence of the beam, the ability of the beamline to resolve the speckle patterns and to record them at a sufficient speed to determine colloidal diffusion coefficients.

The presented experimental results confirmed that the results of the simulations can be used to choose the optimal beam settings for future experiments at the CoSAXS beamline. With the present manuscript we can conclude that the CoSAXS beamline is capable of setting the coherence properties of the delivered beam as it was designed. The coherent beam can be used for XPCS and imaging experiments and soon will be open to the general user community.
